# Combined impacts of histamine receptor H1 gene polymorphisms and an environmental carcinogen on the susceptibility to and progression of oral squamous cell carcinoma

**DOI:** 10.18632/aging.204089

**Published:** 2022-05-19

**Authors:** Yi-Fang Ding, Yung-Wei Lin, Wen-Kuan Chiu, Chiao-Wen Lin, Yi-Chieh Yang, Lun-Ching Chang, Jungshan Chang, Shun-Fa Yang, Ming-Hsien Chien

**Affiliations:** 1 Graduate Institute of Medical Sciences, College of Medicine, Taipei Medical University, Taipei, Taiwan; 2 Department of Otolaryngology, Wan Fang Hospital, Taipei Medical University, Taipei, Taiwan; 3 International Master/PhD Program in Medicine, College of Medicine, Taipei Medical University, Taipei, Taiwan; 4 Department of Urology, Wan Fang Hospital, Taipei Medical University, Taipei, Taiwan; 5 Division of Plastic Surgery, Department of Surgery, Wan Fang Hospital, Taipei Medical University, Taipei, Taiwan; 6 Department of Surgery, School of Medicine, College of Surgery, Taipei Medical University, Taipei, Taiwan; 7 Institute of Oral Sciences, Chung Shan Medical University, Taichung, Taiwan; 8 Department of Dentistry, Chung Shan Medical University Hospital, Taichung, Taiwan; 9 Graduate Institute of Clinical Medicine, College of Medicine, Taipei Medical University, Taipei, Taiwan; 10 Department of Medical Research, Tungs’ Taichung MetroHarbor Hospital, Taichung, Taiwan; 11 Department of Mathematical Sciences, Florida Atlantic University, Boca Raton, FL 33431, USA; 12 Institute of Medicine, Chung Shan Medical University, Taichung, Taiwan; 13 Department of Medical Research, Chung Shan Medical University Hospital, Taichung, Taiwan; 14 Pulmonary Research Center, Wan Fang Hospital, Taipei Medical University, Taipei, Taiwan; 15 Traditional Herbal Medicine Research Center, Taipei Medical University Hospital, Taipei, Taiwan; 16 TMU Research Center of Cancer Translational Medicine, Taipei Medical University, Taipei, Taiwan

**Keywords:** oral squamous cell carcinoma, histamine H1 receptor, single-nucleotide polymorphisms, susceptibility, progression

## Abstract

Oral squamous cell carcinoma (OSCC) is the most frequently encountered type of oral cancer. Histamine receptor H1 (*HRH1*) was reported to play a crucial role in OSCC carcinogenesis, but impacts of genetic variants of *HRH1* on OSCC remain unclear. Herein, we investigated the association between functional single-nucleotide polymorphisms (SNPs) of *HRH1* and OSCC susceptibility or clinicopathologic variables by logistic regression models. *HRH1* genotypes at four loci (rs346074, rs346076, rs901865, and rs2606731) were analyzed by a TaqMan allelic discrimination assay, and we found that patients harboring *HRH1* rs901865 T and rs346074 T alleles had a significantly lower risk of developing larger tumor sizes (>T2) under a dominant model. Based on the environmental carcinogen exposure status, we observed that *HRH1* rs901865 polymorphic variants were also associated with a lower risk of developing more-advanced clinical stages (III or IV) in patients with a betel-quid-chewing habit. Moreover, genotype screening of rs901865 and rs346074 in OSCC cell lines showed that cells respectively carrying the CT and TT genotypes expressed lower HRH1 levels compared to cells carrying the CC genotype of rs901865 and rs346074. Furthermore, analyses of TCGA and GEO databases revealed that *HRH1* expression levels were upregulated in head and neck squamous cell carcinoma (HNSCC) and OSCC tissues compared to normal tissues and were correlated with larger tumor sizes and poorer prognoses. These results indicated the involvement of *HRH1* SNPs rs901865 and rs346074 in OSCC development and support the interaction between *HRH1* gene polymorphisms and an environmental carcinogen as a predisposing factor for OSCC progression.

## INTRODUCTION

Oral cavity cancer, one kind of head and neck cancer, is also one of the most aggressive malignant tumors in the world, with more than 350,000 new cases and over 170,000 deaths in 2018 [1]. The most common histological type of oral cavity cancer is oral squamous cell carcinoma (OSCC), which accounts for more than 90% of cases [2], and the estimated 5-year survival rate of OSCC patients is only 50% [3]. OSCC is the fourth most common cancer in the male population of Taiwan, and the buccal mucosa is the dominant subsite of OSCC due to high betel-quid-chewing rates [4]. Although the combined effects of genetic changes and multiple environmental risk factors were known to trigger OSCC, the causes of OSCC are very complex and a definitive tumorigenesis pathway is yet undefined. Thus, novel predictors for OSCC progression and prognosis, and targets for OSCC therapy still need to be discovered.

Cell proliferation is critical for tumor development and progression, and histamine is a major mediator of these biological processes in different types of cancers [5]. Histamine performs its functions via four histamine receptor subtypes, including histamine receptor H1~H4 (HRH1~HRH4) that belong to the family of G-protein-coupled receptors. Some studies indicated the relevance of histamine receptors to oral potentially malignant disorders (OPMDs) or OSCC. For example, HRH1 was reported to be expressed in OPMDs as well as the BICR56 and BICR3 OSCC cell lines. In OSCC patients, the high HRH1 level was correlated with poor disease-free survival, thus indicating the oncogenic role of HRH1 in OSCC [6, 7]. Parihar et al. indicated that HRH2 level was upregulated in tumor tissues compared to adjacent non-tumor tissues in *in vivo* oral cancer model. They further found that conjugation of histamine with chlorin p6 (Cp6) enhanced cellular uptake and photo toxicity of Cp6 for OSCC [8]. Moreover, HRH2 antagonist treatment in patients with head and neck squamous cell carcinomas (HNSCCs) had significantly better overall survival [9]. HRH4 is expressed by normal oral epithelial cells and is diminished in OSCC, and level of HRH4 was negatively correlated with the OSCC histopathological grade [10]. Taken together, downregulated levels of HRH4 together with upregulated levels of HRH1 and HRH2 may play critical roles in tumorigenesis of oral cancer.

Single nucleotide polymorphisms (SNPs) were identified within genes encoding *HRHs*, and their associations with several diseases including cancers were documented. For example, the GG genotype of rs2607474 (-1018-G/A) in *HRH2* was found to be associated with atrophy of gastric mucosal and the subsequent development of gastric cancer [11]. Moreover, SNPs rs623590, rs11662595, and rs1421125 of *HRH4* were significantly associated with the susceptibility and malignancy of breast cancer. Subjects harboring the rs623590 T allele and rs1421125 A allele respectively had a decreased and an increased risk of developing breast cancer. Furthermore, the haplotype of rs623590-rs11662595-rs1421125 (C-A-A) was found to correlate with risk of breast cancer [12]. Until now, *HRH1* SNP rs346074 was reported to be associated with the treatment efficiency of psychiatric disorders such as bipolar I depression [13]. Moreover, *HRH1* rs901865 SNP was shown to be associated with severe side effect after desloratadine treatment in patients with chronic spontaneous urticarial [14]. Furthermore, Klepstad et al. investigated the impact of *HRH1* SNPs (rs2606731, rs346076, and rs901865) on opioid efficacy in cancer patients [15]. However, compared to the other HRH family members, the impacts of *HRH1* SNPs in cancers have been less well investigated. We hypothesized that functional SNPs of *HRH1* might influence the incidence or progression of OSCC. Therefore, we tried to identify the roles of *HRH1* SNPs in the susceptibility and clinical features of OSCC from a Taiwanese population.

## RESULTS

### General characteristics of study subjects

Table 1 summarizes the basic information including clinicopathological and lifestyle characteristics of all recruited participants. Age frequencies showed no obvious differences between the control and case groups. Compared to the control group, significantly higher frequencies (*p*<0.001) of alcohol consumption, betel-quid chewing, and cigarette smoking were observed in OSCC patients, and these lifestyle characteristics were consistent with Asian OSCC patients reported previously [16, 17], indicating that these lifestyle characteristics should be risk factors of OSCC. The majority of recruited OSCC patients had no lymph node (65.7%) or distal metastasis (99.2%) with moderately/poorly differentiated tumors (85.8%).

**Table 1:** Distributions of demographic characteristics of 1189 controls and 1184 male patients with oral cancer.

**Variable**	**Controls (*N*=1189)**	**Patients (*N*=1184)**	***p* value**
Age (years)			
≤55	605 (50.9%)	591 (49.9%)	0.638
>55	584 (49.1%)	593 (50.1%)	
Betel-quid chewing			
No	993 (83.5%)	311 (26.3%)	
Yes	196 (16.5%)	873 (73.7%)	<0.001*
Cigarette smoking			
No	560 (47.1%)	188 (15.9%)	
Yes	629 (52.9%)	996 (84.1%)	<0.001*
Alcohol consumption			
No	954 (80.2%)	633 (53.5%)	
Yes	235 (19.8%)	551 (46.5%)	<0.001*
Stage			
I+II		558 (47.1%)	
III+IV		626 (52.9%)	
Tumor T status			
T1+T2		583 (49.2%)	
T3+T4		601 (50.8%)	
Lymph node status			
N0		778 (65.7%)	
N1+N2+N3		406 (34.3%)	
Metastasis			
M0		1174 (99.2%)	
M1		10 (0.8%)	
Cell differentiation			
Well differentiated		168 (14.2%)	
Moderately or poorly differentiated		1016 (85.8%)	

The Mann-Whitney U-test or Fisher’s exact test was used to compare healthy controls and patients with oral cancer. * A *p* value of <0.05 was statistically significant.

### Associations of *HRH1* SNPs with OSCC susceptibility

Relationships of four loci of *HRH1* with OSCC susceptibility are presented in Table 2. The four *HRH1* polymorphisms in control group were consistent with Hardy-Weinberg equilibrium (HWE) (χ2 value=0.26, *p*=0.61 for rs346074 C>T, χ2 value=0.25, *p*=0.62 for rs346076 T>C, χ2 value=0.003, *p*=0.96 for rs901865 C>T, and χ2 value=0.10, *p*=0.75 for rs2606731 C>A). Multiple logistic regression models were used to estimate adjusted odds ratios (AORs) (with 95% confidence intervals (CIs)) after adjusting for other variables. We observed no associations between *HRH1* SNPs and OSCC susceptibility in recruited Taiwanese population, as calculated either by a dominant model or a codominant model (Table 2).

**Table 2:** Adjusted odds ratios (AORs) and 95% confidence intervals (CIs) of oral cancer associated with histamine receptor H1 (HRH1) genotypic frequencies.

**Variable**	**Controls (*N*=1189) (%)**	**Patients (*N*=1184) (%)**	**AOR (95% CI)**	***p***
**rs346074**				
CC	502 (42.2%)	501 (42.3%)	1.000 (reference)	
CT	535 (45.0%)	536 (45.3%)	1.061 (0.858~1.313)	0.584
TT	152 (12.8%)	147 (12.4%)	0.959 (0.695~1.322)	0.797
CT+TT	687 (57.8%)	683 (57.7%)	1.038 (0.849~1.270)	0.716
**rs346076**				
TT	471 (39.6%)	470 (39.7%)	1.000 (reference)	
TC	561 (47.2%)	555 (46.9%)	1.043 (0.842~1.293)	0.698
CC	157 (13.2%)	159 (13.4%)	0.936 (0.682~1.284)	0.680
TC+CC	718 (60.4%)	714 (60.3%)	1.019 (0.831~1.249)	0.858
**rs901865**				
CC	1038 (87.3%)	1018 (86.0%)	1.000 (reference)	
CT	146 (12.3%)	156 (13.2%)	1.340 (0.996~1.804)	0.053
TT	5 (0.4%)	10 (0.8%)	1.242 (0.324~4.761)	0.752
CT+TT	151 (12.7%)	166 (14.0%)	1.336 (0.998~1.789)	0.051
**rs2606731**				
CC	582 (49.0%)	592 (50.0%)	1.000 (reference)	
CA	503 (42.3%)	489 (41.3%)	0.963 (0.781~1.186)	0.721
AA	104 (8.7%)	103 (8.7%)	0.817 (0.565~1.179)	0.280
CA+AA	607 (51.0%)	592 (50.0%)	0.936 (0.767~1.142)	0.514

AORs with their 95% CIs were estimated by logistic regression models.

### Associations between *HRH1* genetic variants and clinicopathological characteristics in patients with OSCC

Next, we assessed whether *HRH1* gene polymorphisms were connected to OSCC clinicopathologic features including the primary tumor size, clinical stage, histopathologic grade, and tumor metastatic statuses (Table 3). We observed that OSCC patients harboring one minor allele (CT or TT) of rs346074 showed a significantly lower risk to develop larger tumor sizes (>T2) (AOR: 0.790-fold; 95% CI: 0.626~0.997; *p*=0.048) compared to patients harboring the wild-type (WT) homozygotes (CC). OSCC patients with the CT or TT genotype of rs901865 also showed a lower risk of developing larger tumor sizes (AOR: 0.714-fold; 95% CI: 0.512~0.997; *p*=0.048). In addition to tumor size, no significant association was observed for histological grade, tumor metastasis, or clinical stage of OSCC.

**Table 3:** Adjusted odds ratios (AORs) and 95% confidence intervals (CIs) of clinical statuses associated with genotypic frequencies of histamine receptor H1 (*HRH1*) rs901865 and rs346074 in male oral cancer patients.

**Variable**	**rs901865**				**rs346074**			
	**CC (*N*=1018)**	**CT+TT (*N*=166)**	**AOR (95% CI)^a^**	***p* value**	**CC (*N*=501)**	**CT+TT (*N*=683)**	**AOR (95% CI)**	***p* value**
**Clinical stage**								
Stage I+II	470 (46.2%)	88 (53.0%)	1.000 (reference)	0.086	238 (47.5%)	320 (46.8%)	1.000 (reference)	0.769
Stage III+IV	548 (53.8%)	78 (47.0%)	0.748 (0.537~1.042)		263 (52.5%)	363 (53.2%)	1.035 (0.821~1.306)	
**Tumor size**								
≤T2	490 (48.1%)	93 (56.0%)	1.000 (reference)	**0.048**	231 (46.1%)	352 (51.5%)	1.000 (reference)	**0.048**
>T2	528 (51.9%)	73 (44.0%)	**0.714 (0.512~0.997)**		270 (53.9%)	331 (48.5%)	**0.790 (0.626~0.997)**	
**Lymph node metastasis**								
No	667 (65.5%)	111 (66.9%)	1.000 (reference)	0.620	336 (67.1%)	442 (64.7%)	1.000 (reference)	0.328
Yes	351 (34.5%)	55 (33.1%)	0.915 (0.644~1.300)		165 (32.9%)	241 (35.3%)	1.130 (0.884~1.444)	
**Metastasis**								
M0	1009 (99.1%)	165 (99.4%)	1.000 (reference)	0.639	497 (99.2%)	677 (99.1%)	1.000 (reference)	0.836
M1	9 (0.9%)	1 (0.6%)	0.607 (0.075~4.880)		4 (0.8%)	6 (0.9%)	1.144 (0.320~4.092)	
**Cell differentiated grade**								
Well	143 (14.0%)	25 (15.1%)	1.000 (reference)	0.573	68 (13.6%)	100 (14.6%)	1.000 (reference)	0.602
Moderately or poorly	875 (86.0%)	141 (84.9%)	0.875 (0.550~1.392)		433 (86.4%)	583 (85.4%)	0.915 (0.655~1.277)	

Cell differentiate grade: grade I, well differentiated; grade II, moderately differentiated; grade III, poorly differentiated.

^a^The AORs with their 95% CIs were estimated by multiple logistic regression models after controlling for age, betel-quid chewing, cigarette smoking, and alcohol consumption.

### Combined and interactive effects of *HRH1* SNPs and betel-quid chewing on OSCC progression

In East Asia, including Taiwan, betel-quid chewing is already known to be a dominant risk factor for OSCC and has been correlated with poor survival of OSCC patients [18–20]. Herein, we separated the recruited patients into subpopulation patients with and without betel-quid-chewing habits, and further investigated differences between *HRH1* SNPs and the OSCC clinicopathological features in these two subpopulations. The significantly lower risk of developing an advanced clinical (III+IV) stage was observed in OSCC patients who chewed betel quid and also carried at least one polymorphic T allele of rs901865 (AOR: 0.614-fold; 95% CI: 0.408~0.924; *p*=0.020) (Table 4). In contrast, in subpopulation patients without the habit of betel-quid-chewing, the protective effect of rs901865 polymorphisms was not observed (Table 5). Taken together, our results indicated that a potential interaction between the existence of at least one minor allele of an *HRH1* rs901865 SNP and betel-quid chewing was shown to be associate with OSCC progression.

**Table 4:** Adjusted odds ratios (AORs) and 95% confidence intervals (CIs) of clinical statuses associated with genotypic frequencies of histamine receptor H1 (*HRH1*) rs901865 in betel-quid chewers.

**Variable**	**Betel-quid chewers (*N*=873)**			
	**CC (*N*=764)**	**CT+TT (*N*=109)**	**AOR (95% CI)^a^**	***p* value**
**Clinical stage**				
Stage I+II	354 (46.3%)	64 (58.7%)	1.000 (reference)	**0.020**
Stage III+IV	410 (53.7%)	45 (41.3%)	**0.614 (0.408~0.924)**	
**Tumor size**				
≤T2	376 (49.2%)	63 (57.8%)	1.000 (reference)	0.131
>T2	388 (50.8%)	46 (42.2%)	0.730 (0.485~1.098)	
**Lymph node metastasis**				
No	503 (65.8%)	79 (72.5%)	1.000 (reference)	0.163
Yes	261 (34.2%)	30 (27.5%)	0.726 (0.463~1.139)	
**Metastasis**				
M0	757 (99.1%)	109 (100.0%)	-	-
M1	7 (0.9%)	0 (0.0%)		
**Cell differentiated grade**				
Well	120 (15.7%)	16 (14.7%)	1.000 (reference)	0.740
Moderately or poorly	644 (84.3%)	93 (85.3%)	1.101 (0.624~1.943)	

Cell differentiate grade: grade I, well differentiated; grade II, moderately differentiated; grade III, poorly differentiated.

^a^The AORs with their 95% CIs were estimated by multiple logistic regression models after controlling for age, cigarette smoking, and alcohol consumption.

**Table 5:** Adjusted odds ratios (AORs) and 95% confidence intervals (CIs) of clinical statuses associated with genotypic frequencies of histamine receptor H1 (*HRH1*) rs901865 in non-betel-quid chewers.

**Variable**	**Non-betel-quid chewers (*N*=311)**			
	**CC (*N*=254)**	**CT+TT (*N*=57)**	**AOR (95% CI)^a^**	***p* value**
**Clinical stage**				
Stage I+II	116 (45.7%)	24 (42.1%)	1.000 (reference)	0.582
Stage III+IV	138 (54.3%)	33 (57.9%)	1.179 (0.656~2.121)	
**Tumor size**				
≤T2	114 (44.9%)	30 (52.6%)	1.000 (reference)	0.249
>T2	140 (55.1%)	27 (47.4%)	0.709 (0.396~1.272)	
**Lymph node metastasis**				
No	164 (64.6%)	32 (56.1%)	1.000 (reference)	0.189
Yes	90 (35.4%)	25 (43.9%)	1.485 (0.823~2.679)	
**Metastasis**				
M0	252 (99.2%)	56 (98.3%)	1.000 (reference)	0.412
M1	2 (0.8%)	1 (1.7%)	2.798 (0.240~32.609)	
**Cell differentiated grade**				
Well	23 (9.1%)	9 (15.8%)	1.000 (reference)	0.151
Moderately or poorly	231 (90.9%)	48 (84.2%)	0.542 (0.235~1.251)	

Cell differentiate grade: grade I, well differentiated; grade II, moderately differentiated; grade III, poorly differentiated.

^a^The AORs with their 95% CIs were estimated by multiple logistic regression models after controlling for age, cigarette smoking, and alcohol consumption.

### Upregulation of *HRH1* in OSCC tissues correlates with tumor progression and a poor prognosis

**Figure 1:**
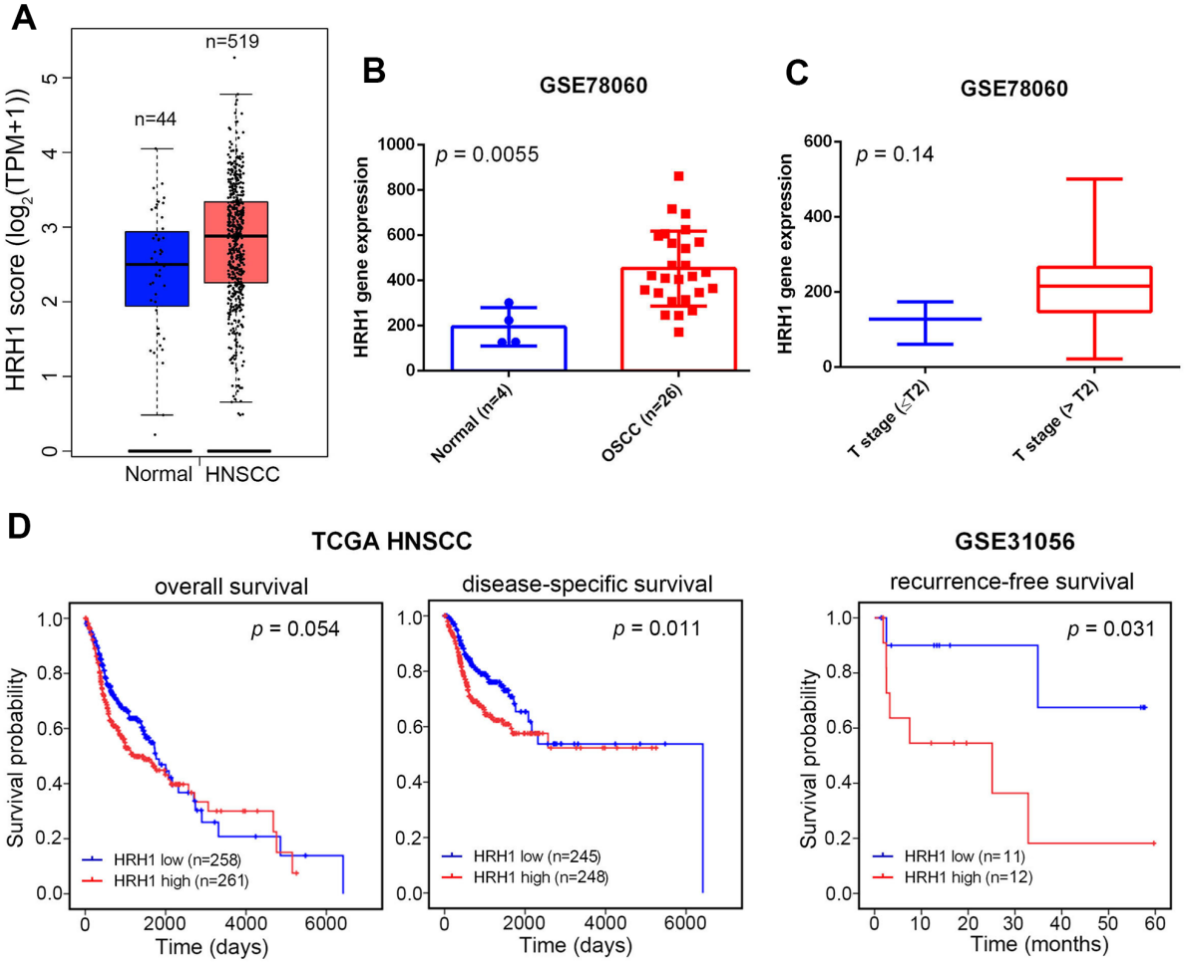
**Clinical relevance of histamine receptor H1 (HRH1) levels in head and neck squamous cell carcinoma (HNSCC) or oral squamous cell carcinoma (OSCC) patients obtained from TCGA and GEO databases.** (**A**, **B**) HRH1 expression was higher in HNSCC tissues (**A**) and OSCC tissues (**B**) than in normal tissues (database sources: TCGA and GSE78060). (**C**) HRH1 expression levels in OSCC from GSE78060 were compared according to the tumor size (T stages). (**D**) Kaplan-Meier curves for overall and disease-specific survival (left panel) and recurrence-free survival (right panel) of patients with HNSCC and OSCC, as categorized according to high or low expression of *HRH1*. The *p* value indicates a comparison between patients with HRH1^high^ and HRH1^low^. (database sources: TCGA and GSE31056).

We further analyzed correlations of *HRH1* expression levels and their clinical significance or survival rates in oral cancer, by examining cases of HNSCCs from The Cancer Genome Atlas (TCGA) dataset. We observed that *HRH1* expression was prone to be upregulated in HNSCCs compared to noncancerous tissues (Figure 1A). To focus on OSCC, we analyzed dataset (GSE78060) from Gene Expression Omnibus (GEO) database and also found that *HRH1* expression levels were significantly higher in OSCC tissues compared to normal tissues (Figure 1B). In the same GSE cohort, we observed that *HRH1* transcripts were prone to be higher in OSCC patients with larger tumors (>T2 status) than in patients with smaller tumors (≤T2 status) (Figure 1C). Moreover, a Kaplan-Meier plot revealed that HNSCC patients from TCGA with HRH1^high^ tumors had shorter overall and disease-specific survival times compared to those with HRH1^low^ tumors (*p*=0.054 and 0.011; Figure 1D, left panel). Furthermore, 23 OSCC cases from the GEO database (GSE31056) were also analyzed, and shorter recurrence-free survival times was observed in patients harboring HRH1^high^ tumors compared to those harboring HRH1^low^ tumors (*p*=0.031; Figure 1D, right panel). Altogether, these data indicated that *HRH1* SNPs might influence *HRH1* expression and subsequently modulate OSCC progression and contribute to poor prognoses.

### Genotype-based *HRH1* expression analysis

**Figure 2:**
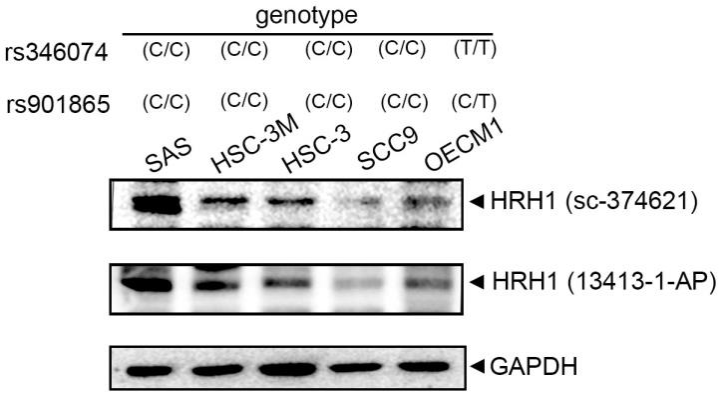
**Correlations of histamine receptor H1 (HRH1) rs346074 and rs901865 genotypes with HRH1 protein levels in five oral squamous cell carcinoma (OSCC) cell lines.** Upper panel, HRH1 rs346074 or rs901865 genotypes in OSCC cells (SAS, HSC-3M, HSC-3, SCC9, and OECM1) were detected by a TaqMan SNP Genotyping Assay. Lower panel, protein levels of HRH1 were detected by a Western blot analysis using two HRH1-specific antibodies.

To further investigate correlations of *HRH1* rs901865 and rs346074 polymorphisms with HRH1 expression levels in OSCC, we examined rs901865 or rs346074 genotypes of five OSCC cell lines (SCC9, HSC-3, HSC-3M, SAS, and OECM1) and found that OECM1 cells carried the CT genotype of rs901865 and the TT genotype of rs346074 compared to HSC-3M, SAS, and HSC3 cells which carried the CC genotype of both SNPs (Figure 2, upper panel). Moreover, we detected HRH1 expression by two different antibodies using a Western blot analysis. Among these OSCC cell lines, we observed that OECM1 cells expressed lower HRH1 protein levels than SAS, HSC-3M, or HSC-3 cells (Figure 2, lower panel).

## DISCUSSION

Histamine and its receptor, HRH1, are known to play important roles in many aspects of cancer development including cell proliferation [5, 21]. Recently, high expression levels of histamine or HRH1 were observed in different human cancers and were correlated with poor prognoses including in breast [22, 23], colon [24], and liver cancers [25, 26]. In the current study, we found that *HRH1* was upregulated in OSCC and was correlated with larger tumor sizes and poor prognoses of OSCC patients, suggesting an oncogenic role of HRH1 in OSCC. Previous studies indicated that OSCC is a disease related to multiple gene mutations [27], and some of the gene polymorphisms were reported to be associated with OSCC risks and progression [28, 29]. Although previous reports indicated that *HRH1* messenger (m)RNA expression was elevated in subjects with asthma and genetic variants of *HRH1* contributed to the risk of allergic asthma [30, 31], knowledge of the clinical relevance of *HRH1* SNPs in OSCC, which probably result in expression and functional changes of HRH1, is still lacking. Herein, we found for the first time that polymorphisms of the *HRH1* gene play critical roles in influencing the clinicopathological characteristics of OSCC in a Taiwanese population.

Our present data indicated that patients with a mutant base T of rs346074 or rs901865 had a significantly reduced risk of developing larger tumor sizes (>T2) under a dominant model (CT+TT). Previous *in vitro* and *in vivo* investigations showed that agonists of HRH1 such as histamine significantly induced progression (growth and metastasis) of liver cancer via HRH1 which was highly expressed in liver cancer tissues [25]. Previous studies indicated that some SNPs of *HRHs* were shown to influence their mRNA expression levels. For example, the SNPs ss142022671 and ss142022677 of *HRH4* were respectively reported to increase the *HRH4* mRNA level and protein stability [32]. Actually, Li et al. indicated that Chinese patients with chronic spontaneous urticaria who harbor the *HRH1* rs901865 CC polymorphism were associated with severe sedation side effects caused by desloratadine treatment. They claimed that patients harboring rs901865 CC genotype had high levels of *HRH1* transcripts, which triggered serious sedation side effects [14]. In the same race, Chu also indicated that patients harboring the CC genotype of rs901865 exhibited the increased response of H1-antihistamines treatment in patients with allergic rhinitis [33]. In contrast, in African American populations with asthma, no difference in *HRH1* mRNA expression was detected relative to the rs901865 genotype [34], suggesting that the *HRH1* rs901865 polymorphism contributing to *HRH1* gene expressions might be diverse in different races. In addition to rs901865, the rs346074 SNP located on transcription factor binding sites of the *HRH1* gene may affect transcription rates [35], but the impact of rs346074 SNPs on *HRH1* mRNA expression should be further investigated. Actually, our present study further examined the genotypes of rs901865 and rs346074 in five OSCC cell lines (SCC9, HSC-3, HSC-3M, SAS, and OECM1) and found that OECM1 cells carrying the rs901865 CT genotype and the rs346074 TT genotype expressed lower HRH1 protein levels compared to SAS, HSC-3, and HSC-3M cells carrying the CC genotype of both SNPs, suggesting that the T allele of rs901865 or rs346074 may produce a decrease in *HRH1* levels in OSCC and subsequently cause smaller tumor sizes in OSCC patients carrying one minor allele of rs901865 or rs346074.

Because betel-quid chewing increased the risk of OPMDs and OSCC [36, 37], herein, we explored the combined effect of *HRH1* SNPs and betel-quid chewing on OSCC progression. We observed that patients with one T allele of *HRH1* rs901865 had a lower risk to develop an advanced clinical stage in the subpopulation of betel-quid chewers, and this phenomenon was not observed in the subpopulation who did not chew areca nut. Previous studies showed that betel-quid chewing can induce histamine release and further cause gastric hemorrhaging and mucosal ulceration in rats. Activation of HRH1 is involved in aggravating histamine-induced gastric mucosal ulceration in betel-quid-fed rats, and this phenomenon was ameliorated by treatment with the antihistamine, ketotifen [38]. We suggest that betel-quid chewing might induce upregulation of histamine in OSCC patients with a betel-quid-chewing habit and promote OSCC progression via HRH1, and the protective effect observed in OSCC patients carrying the CT/TT genotypes of *HRH1* rs901865 might be due to lower expression levels of *HRH1* in this subgroup.

Histamine and its receptors play critical roles in several cancer-associated processes in tumor microenvironment, where mast cells (MCs) is a major source of histamine. Previous reports indicated that numbers of MCs are higher among OPMDs and OSCC compared to normal oral mucosa [39]. The MCs mainly located at the lamina propria surrounding the tumor invasive front of OSCC. Increased MCs were also observed in hotspot microvascular density areas, suggesting MC numbers may correlate with angiogenesis [40–42]. Based on the previous studies, the upregulated levels of HRH1/HRH2 and tumor-associated MCs may play crucial roles in promoting tumorigenesis and progression of OSCC [7]. Therefore, the correlation among *HRH1* SNPs, MCs infiltration, and OSCC progression will be further investigated in our future work.

Nevertheless, this study still has some limitations that need to be discussed. First, all of the subjects included in this study were Taiwanese (of Chinese ethnicity), so it is unclear whether these results can be observed in other races. Therefore, further investigations of these SNPs in different racial OSCC populations are necessary to confirm our results. Second, the sample size was not large enough, which might cause statistical impacts on the accuracy of the results. Third, the functional impacts of *HRH1* SNPs on OSCC is still unclear. Moreover, we should simultaneously collect DNA and mRNA from the same samples to check the influences of *HRH1* SNPs on *HRH1* expression in future. Last, this study just focused on the association of *HRH1* SNPs with OSCC, and the impacts of other genetic variations within the histamine pathway including those of L-histidine decarboxylase, histamine N-methyltransferase, and diamine oxidase on OSCC need to be further investigated.

In summary, our study first pointed out the diverse allelic effects of *HRH1* SNPs (rs901865 and 346074) which affect the clinicopathologic features of OSCC. Moreover, the combined effect of rs901865 SNPs with betel-quid chewing was found to causally influence the development of OSCC. Our preliminary analysis indicated that the T allele of rs901865 or rs346074 may cause a decrease in HRH1 levels in OSCC cells. At present, a large proportion of OSCC patients still will develop advanced disease eventually, and the reliable tools are lacking to predict who those patients are. Our present results provide novel information for detecting high-risk populations for OSCC and formulating individualized preventive measures.

## MATERIALS AND METHODS

### Study population selection

Between 2010 and 2021, a total of 1184 male patients diagnosed with OSCC were randomly collected from a patient database of Chung Shan Medical University Hospital (Taichung, Taiwan). In addition, 1189 anonymized healthy male controls without a history of oral precancerous disease or other cancers were randomly selected from the Taiwan Biobank Project. All study populations were Han Chinese and lived in the same geographic area. Medical information and demographic data, including age, gender, primary tumor size, the tumor, node, metastasis (TNM) clinical staging, lymph node and distal metastasis, and histologic grade, were all obtained from medical records. The protocol of this case-control study was approved by the Ethics Committee of Chung Shan Medical University Hospital (no. CS1-21151).

### OSCC cell lines and culture

The human SAS, HSC-3, HSC-3M, and SCC9 OSCC cell lines were purchased from the Japanese Collection of Research Bioresources Cell Bank (JCRB, Osaka, Japan) or the American Type Culture Collection (ATCC, Manassas, VA, USA). All four of these OSCC cell lines were cultured in Dulbecco’s modified Eagle medium/nutrient mixture F-12 (DMEM/F12; Life Technologies, Grand Island, NY, USA) containing 10% fetal bovine serum (FBS) (Gibco, Grand Island, NY, USA). The OECM1 OSCC cell line derived from surgical resection of a primary tumor from a male Taiwanese patient [43] was maintained in RPMI-1640 medium (Life Technologies) with 10% FBS. All cells were maintained in an incubator (37° C, 5% CO_2_ and 95% air atmosphere).

### DNA extraction and SNP genotyping for allelic frequencies of the *HRH1* gene

Genomic DNA was isolated from peripheral blood leukocytes of all participants or OSCC cell lines using a Genomic DNA Extraction kit (Qiagen, Valencia, CA, USA) according to the manufacturer’s instructions. The DNA concentration was detected with a Nanodrop-2000 spectrophotometer (ThermoFisher Scientific, Waltham, MA, USA) and stored at -20° C before genotyping. Allelic discrimination of the four *HRH1* SNPs including rs346074 (assay ID: C__26855885_10), rs346076 (assay ID: C__11984711_10), rs901865 (assay ID: C__25471612_10), and rs2606731 (assay ID: C__15902251_10), was determined by the TaqMan SNP Genotyping Assay which utilized the ABI StepOnePlus™ Real-Time PCR System (Applied Biosystems, Foster City, CA, USA) and ABI StepOnePlus™ Software (version 2.3) used to analyze the final data.

### *In silico* expression analyses of the human *HRH1* gene

The Gene Expression Profiling Interactive Analysis (GEPIA) (http://gepia.cancer-pku.cn/index.html) online database [44] is a communal network server that includes a transcriptome sequencing dataset of 9736 tumors together with 8587 adjacent normal tissues from Genotype-Tissue Expression (GTEx) and The Cancer Genome Atlas (TCGA) datasets. Herein, we compared the expression of *HRH1* between HNSCC and normal tissues. Moreover, transcriptomic data of normal and OSCC tissues from an Asian population was also analyzed using the GSE78060 dataset from the GEO database (https://www.ncbi.nlm.nih.gov/geo/). The prognostic significance of HRH1 in HNSCC and OSCC was determined using a Kaplan-Meier analysis of TCGA and GEO databases, respectively.

### Total protein extraction and Western blot analysis

Total cell lysate extraction was carried out and protein contents were determined as previously described [45]. A Western blot analysis was performed with primary antibodies for HRH1(sc-374621, Santa Cruz Biotechnology, Heidelberg, Germany; 13413-1-AP, Proteintech Group, Chicago, IL, USA) or GAPDH (#2118, Cell Signaling Technology, Danvers, MA, USA).

### Statistical analysis

Differences in demographic parameters between OSCC patients and cancer-free controls were analyzed using the Fisher’s exact test and Mann-Whitney *U*-test. Using multiple logistic regression models after controlling for covariates, the AORs with their 95% CIs for the association between genotype frequencies and the OSCC risk or clinical pathological characteristics of OSCC were calculated. Statistical analysis of our data used the SAS software program (version 9.1, 2005; SAS Institute, Cary, NC, USA). A *p* value of <0.05 was considered significant.
